# Camp Douglas

**Published:** 1862-08

**Authors:** 


### ORIGINAL COMMUNICATIONS.

### CAMP DOUGLAS.

On the 26th of June the order was carried into effect releasing the rebel Surgeons confined in Camp Douglas. The order was as grateful to the profession as it was to the released Surgeons, marking as it does a new era for the noble of our number who have gone to succor and to save our patriot soldiers. Henceforth no Surgeons are to be taken prisoners either by ours or the rebel army. Nothing could be more satisfactory than this wise, humane and judicious arrangement. It was always a mystery to me, why Surgeons were classed with the soldiery and treated and held as prisoners of war. They are strictly non-combatants, whose sole end, aim and desire is to save and not destroy, help and not harm.

It is also well known that the Surgeons of both armies are as faithful to the needy of the enemies as their own, letting their profession, as it should, raise them above the bitter strife, and fulfilling their noble mission with philanthropic devotion. Surgeon prisoners thus give no strength to those who capture, nor weaken those who lose them, and there seems, therefore, no inducement to retain them. It will induce many valuable Surgeons to volunteer who could not consent to the risks involved in the past rules of our warfare. The truly conscientious and true Surgeon when once in the field and in charge of his wounded would not be deterred by the approach of the enemy from the quiet continuance of his responsible duties, though the next minute found him a prisoner with all its horrible realities. The first duty of a man in such a situation is to those who have trusted their lives to him, and to whom moments of prompt labor may be everything. I don't think some eminent Surgeons who so gloriously fled from the

Bull's Run wounded felt well when they thought of the unmanly, cowardly, treacherous and inhuman part they had acted, even if they were safe in Washington. Their parting address to 250 wounded under their care, "Thank God, boys, you are not much hurt, and I think you will all get well," must have been very grateful to suffering and dying men, and brought that comforting response, "Thank you, Dr.—thank you," though they confess they were ashamed to tell the helpless men they were going. No subsequent approvals of Major Generals has ever made his Eminence satisfied with the unfeeling part he acted in the desertion of his brave and doubly unfortunate dependents. The probability of death would have been an additional reason why he ought to have stayed. Such necessities, I am proud to say, do not now exist, and Surgeons dare do their duty with no fear of months of imprisonment and suffering, as a penalty for their faithfulness.

The rebel Surgeons released numbered nineteen, viz:

Joseph Sandeck, Surgeon Heavy Artillery.
Sam'l H. Caldwell, Surgeon 46th Tennessee.
Thos. J. Taliaferro, Ass't Surgeon 41st Tennessee.
Delmos F. Connell, Ass't Surgeon 1st Alabama.
J. McLin Driver, Surgeon 55th Tennessee.
Mathew H. Oliver, Ass't Surgeon 17th Alabama.
Robert H. Redwood, Surgeon 21st Alabama.
Caleb Foxey, Ass't Surgeon 19th Alabama.
Elisha G. Greenlee, Surgeon 2d Kentucky.
Robert G. Rethrock, Ass't Surgeon 2d Kentucky.
John F. Kennedy, Surgeon 14th Mississippi.
Kelly Williams, Ass't Surgeon 14th Mississippi.
John F. McDowell, Ass't Surgeon 12th Alabama.
Robt. A. Fulton, Ass't Surgeon 7th Texas.
Thos. B. Elkin, Ass't Surgeon 20th Mississippi.
William A. Martin, Surgeon 1st Ala., Tenn. and Miss.
Michael J. Bolar, Surgeon 17th Alabama.
Sam'l B. Johnston, Ass't Surgeon, Regular C. S. A.
Jas. W. Duprer, Ass't Surgeon Light Artillery.

They were occupied in attending to the wants of the prisoners confined in the Camp. In the main they *did* well, though some of them seemed very indifferent to the sufferings of their comrades, and but one refused to do service, and that one gave way before the prospect of the "White Oaks;" though I fear if he had been put in he never would have *seen* his folly. There are several physicians still left in the Camp who are not regularly in the rebel service as such, and to such, of course, the order did not apply, though all who performed the offices of surgeons, whether regularly commissioned, were liberally released by Col. Tucker, as they should have been.

The absence of the rebel Surgeons rendered a supply necessary for filling their places. The following were appointed and began service on the 26th of June, as "Contract" Surgeons, as they are termed: Dr. Chas. G. Smith, Dr. M. O. Heydock, Dr. J. A. Hahn, Dr. Ross and Dr. R. L. Rea, all residents of this city. In addition, three young men, under graduate, were appointed: Frand Mehler, E. E Lynn and Henry Pierce.

Each of the physicians named have assigned them wards, to which they give exclusive attention, and are answerable to the Surgeon of the Post for its good management. The young men attend to Barrack duty; that is, many are taken with slight indispositions which are not severe enough to send them to the Hospitals and yet need to be treated promptly to anticipate worse conditions. They are under the supervision of the attending Surgeons, to whom they report daily after visiting their different barracks.

The rebel attendants are not assigned any duty, though they make themselves useful in prescribing through the different barracks, as their inclination leads them. The compounding prescriptions is performed by themselves, by young men found among them who have a knowledge of pharmacy, many of them being practical druggists. We furnish one principal druggist who superintends this department.

Two regiments of Infantry, enlisted for the three month's service, act as guards. Sixty-Ninth, Col. Tucker, Commandant of Post; Dr. Brock McVickar, Post Surgeon; Assistant Dr. Hale; and the Sixty-Seventh, Col. Hough; Surgeon, Dr. J. P. Lynn; Asst., Dr. —— Goodwin. The Surgeons of these regiments confine themselves to their own sick; the duties of Dr. McVickar as Post Surgeon necessarily occupy him so entirely as to prevent his attendance on his regiment, and throws the duties on the other Surgeons.

The number of sick varies from three to four hundred in the hospitals and from two to three hundred in the barracks. There were in the hospital lists at the beginning of my term of service three hundred and twenty.

The character and variety of diseases change little. Diseases affecting the lungs and bowels, with Typhoid Fever, comprehends the list. The usual termination is about the same—death. The Grim General has recruited almost a regiment from the shattered bodies of rebel sick. The mortality is frightful. From five to twelve "fall in" every day. In my wards, containing near eighty, I contribute on a daily average only two or three; yet in some of the wards I understand they die *very fast!*

This fearful mortality can be accounted for in the usual effects of the soldier's mode of living, and in addition, in the cases of these prisoners, the change of climate. The Camp is as well ordered as it is possible to keep it; and under the efficient management of Col. Tucker, a system of drainage is being inaugurated which will undoubtedly tell on the health of the prisoners. The prisoners themselves are made to dig drains through the various parts of the Camp, directed to the lake, which will prevent the flooding of the Camp and the necessary mud. Damp, combined with cold, is the great exciting cause of disease among them. In a camp situated as this is, where water settles about in quantities and where cold or cool weather prevails, combined with the carelessness of themselves, and then let these causes act on persons used to a warm climate, and we have a key to the extraordinary amount of disease prevailing. Many of these affected have been sick ever since they were taken at Donelson. We remember the cold and rainy weather prevailing at the time of its surrender; the prisoners were huddled together on the transports, very many without blankets, and compelled to stay the entire time from the Fort to St. Louis on the hurricane deck, exposed to storm and cold, which has laid the foundation for disease from which they will never recover. Soldiers are habitually careless of themselves. They seem to lose all anxiety for their own health when they enlist, and three-fourths of the sickness which we find among them is chargeable to their own folly. A soldier on guard gets his clothing saturated with rain, and when released, goes into tent throws his wearied body down, and, without the change of an article, sleeps. Another, with diarrhœa or dysentery, tired of his camp diet, lives on sutler's pies or half-cooked beans and bacon, and thus makes often incurable, by his own neglect and folly, what would be a very manageable malady.

One thing has struck the attention of all who have seen much of army diseases: their depressed and adynamic form. They very speedily assume, if they are not originally, a low grade. We see men who have every appearance of robust and vigorous constitutions dying of diseases and injuries which in civil practice would be considered easily curable. In amputation for moderate injuries we can have no very positive assurance of a favorable result. One of the best marked instances which has ever occurred under my notice took place in the Camp. One of our own soldiers was wounded by a rail car passing over the outside of his foot—not a severe injury by any means; mortification set in. I amputated his leg below the knee. He had every appearance of being a good subject for the operation; pulse good, fine spirits, and I had no apprehensions as to the result. The wound never made the most remote effort at healing; stitches tore out, wound dropped open, point of the flaps mortified, erysipelas confined to the flaps set in, with secondary hemorrhage. I re-amputated in the middle of the thigh, and applied Tinc. Iron thoroughly to the flaps. This time they seemed for two or three days as though going to heal, but he sank and died at the end of the fourth day. After each operation he was put on nourishing broths, with Iron, Quinine and stimulants.

Very many die of consumption. The depressed and vitiated condition of their systems facilitate its development.

Within the last few weeks Scurvy has made its appearance, to add to the complications of cure. It is a difficult matter to control Diarrhœa and Scurvy where they occur in the same individual. The free use of fresh vegetables, which are the only help in Scurvy, aggravating the irritation of the bowels, and render their administration many times worse than useless. In such cases we use vegetable soup, in moderate quantities, with good effect, and, when the diarrhœa is not very severe, the vegetables themselves. Scurvy no doubt makes itself felt on all the prevailing diseases, Hæmorrhage from the mucus membranes being vastly more common since its appearance. As in Dysentery and Pneumonia, the moderate discharges of blood are replaced by more free ones. The occurrence of Scurvy has its usual cause of a lack of fresh vegetables. Before fresh vegetables can be obtained at reasonable prices, and just as last year's potatoes are disappearing from the market, we have the natural time for its occurrence. If potatoes were liberally used I don't think Scurvy would ever occur. We urge the free use of them, with cabbages, which are now plentiful, and the disease very readily yields. We use in these cases, also, Tr. Iron internally three times daily. There is one use of the Tinc. Iron which I regard as invaluable, and that is in its local application to the gums. In almost all these cases the gums are swollen, tender and bleeding, rendering it exceedingly painful to masticate, in this way offering an obstacle to a speedy cure. The Tinc. of Iron has an almost magical influence in curing them. I first used it in the Commercial Hospital in Cincinnati, 1855, after trying

Nitr. Silver, Sugar of Lead, Alum, &c., and was delighted at the surprising and sudden change it would produce—many of the very worst cases being cured in four or five days. I apply it pure to the gums, inside and out, with a camel's hair brush, thoroughly three times a day. It is rather disagreeable, but patients do not long object to it. It is amost as effectual in cases of salivation as scurvy, though not quite so prompt. We use also lemons and Citric Acid drinks. Chlorate of Potash has been somewhat used, but it seems to add but little to the haste of cure, if any.

The influence of Scurvy in this, as I have noticed before, seems to show itself in a susceptibility to the effects of Mercury. One of the vilest salivations I have ever seen occurred to a man who came into my ward, caused by the application of the patient to himself of a small amount of Ung. Hyarg. for body lice. He had never been ptyalized before, and so great was the swelling of his face and tongue that he has not spoken for near two weeks.

